# The silencing of indoleamine 2,3-dioxygenase 1 (IDO1) in dendritic cells by siRNA-loaded lipid nanoparticles enhances cell-based cancer immunotherapy

**DOI:** 10.1038/s41598-019-47799-w

**Published:** 2019-08-05

**Authors:** Rikito Endo, Takashi Nakamura, Kyoko Kawakami, Yusuke Sato, Hideyoshi Harashima

**Affiliations:** Faculty of Pharmaceutical Sciences, Hokkaido University, Kita-12, Nishi-6, Kita-ku, Sapporo, 060-0812 Japan

**Keywords:** Nanoparticles, RNAi therapy, Cancer immunotherapy

## Abstract

Cell-based therapy using dendritic cells (DC) represents a potent cancer immunotherapy. However, activated DC express indoleamine 2,3-dioxygenase 1 (IDO1), a counter-regulatory and tolerogenic molecule, leading to the inhibition of T cell activation and the promotion of T cell differentiation into regulatory T cells. Silencing the IDO1 gene in DC by small interfering RNA (siRNA) represents a potent therapeutic strategy. We report on the successful and efficient introduction of a siRNA targeting IDO1 into mouse DCs by a means of a multifunctional envelope-type nanodevice (MEND) containing a YSK12-C4 (YSK12-MEND). The YSK12-C4 has both fusogenic and cationic properties. The YSK12-MEND induced an effective level of gene silencing of IDO1 at siRNA doses in the range of 1–20 nM, a concentration that commercially available transfection reagents are not able to silence. The YSK12-MEND mediated IDO1 silencing had no effect on the characteristic determinants of DC phenotype such as CD11c, CD80 and MHC class II. The silencing of IDO1 in DC by the YSK12-MEND significantly enhanced the antitumor effect against E.G7-OVA tumor. Moreover, a decrease in the numbers of regulatory T cells in the tumor was observed in mice that were treated with the IDO1-silenced DC. The YSK12-MEND appears to be a potent delivery system for IDO1-silenced DC based cancer immunotherapy.

## Introduction

Dendritic cells (DCs) are one of the most powerful antigen presenting cells (APCs). The engulfment of antigens and the cross-presentation of antigens by DCs represents a critical bridge between innate and adaptive immunity. In cancer, the sensing of cancer cells or tumor antigens by DCs stimulates a sequence of anti-tumor immune responses^1,2^. Thus, DC-based therapy represents a potent approach in cancer immunotherapy and this approach has been extensively developed. The first FDA-approved cell-based therapy, Provenge (Sipuleucel-T), was composed of activated blood mononuclear cells including DCs, and was used for the treatment of prostate cancer. However, this approach was a subject of criticism, because the effect was not as strong as would be expected^3^. The cause of the modest efficacy can be attributed to the immune suppressive characteristics of the tumor-associated microenvironment. It is now well-known that effector immune cells such as DCs, cytotoxic T cells (CTLs), helper T cells are functionally suppressed by several mechanisms such as a loss of immunogenicity, immune check point molecules, immune suppressive molecules, regulatory T (Treg) cells and myeloid-derived suppressor cells (MDSCs)^4,5^. Given this situation, DC-based therapy combined with the inhibition of immune suppression are currently being investigated in preclinical and clinical investigations^6^. On the other hand, stimulation by adjuvants or cytokines leads to the upregulation of counter-regulators, such as the suppressor of cytokine signaling 1 (SOCS1), A20 and indoleamine 2,3-dioxygenase-1 (IDO1), in immune responses in DCs^7–10^. Therefore, the inhibition of these counter-regulators represents a promising strategy for enhancing the efficacy of DC-based therapy^11–15^.

Increasing evidence indicates that IDO1, an enzyme associated with tryptophan metabolism, greatly contributes to the immune suppression in a tumor-associated microenvironment^16^. IDO1 activity is upregulated in many types of human cancer and tends to be associated with a poor prognosis^17^. The depletion of tryptophan by IDO1 leads to the suppression of effector T cells, the differentiation of naïve T cells to Treg cells, the functional promotion of MDSCs and the recruitment of tumor vasculature^18^. IDO1-expressing DCs induce several types of negative regulation including the inhibition of T cell priming and proliferation, and the differentiation of naïve T cells to Treg cells^10,19^. Therefore, the inhibition of IDO1 in DCs is considered to be a promising strategy for enhancing the efficacy of DC-based therapy, because the activation of DCs by adjuvants or cytokines *in vitro* promotes the expression of IDO1.

Gene silencing by small interfering RNA (siRNA) is powerful and straightforward approach for inhibiting the expression of target genes *in vitro*. DCs in which IDO1 is silenced by siRNA show enhanced immuno-stimulative activities and the efficacy of DC-based therapy is enhanced against cancer^14,20^. In these reports, however, high levels of siRNA (2 μg) or electroporation methodology were used to introduce the siRNA into DCs. The requirement of a high dose and special equipment detracts from the potentially broad utility and practical application of this approach. Thus, a delivery system that can efficiently introduce siRNA into DCs, is easily handled, and does not require special equipment, would be highly desirable in this regard. Although transfection reagents for delivering siRNA into cells are in widespread use, some of which are commercially available (Lipofectamine® RNAiMAX (RNAiMAX), X-tremeGENE, ViaFect^TM^, etc), delivering siRNA to immune cells, for example DCs, T cells, B cells, natural killer (NK) cells and macrophages, by these commercially available reagents is quite difficult.

In the previous studies, we designed a multifunctional envelope-type nanodevice (MEND) for use as a non-viral delivery system for nucleic acids^21–23^ and we developed a MEND containing the YSK12-C4 lipid (YSK12-MEND) that can be used to deliver siRNA to several types of immune cells^15,24,25^. YSK12-C4 is a cationic lipid containing unsaturated carbon chains, which enhances endosomal escape. The YSK12-MEND resulted in a 90% gene silencing activity with a median effective concentration (EC_50_) of 1.5 nM in DC. On the other hand, the EC_50_ for RNAiMAX was 25 nM^15^. In addition, the DCs in which SOCS1 had been silenced by the YSK12-MEND showed drastically enhanced immuno-stimulative activities and the efficacy of cell therapy using the SOCS1-silenced DC in tumor bearing mice was increased substantially. These findings indicate that the YSK12-MEND represents a promising delivery system that can result in effective gene silencing using a low dose of siRNA and is easily handled.

We report here on confirming the potency of the YSK12-MEND in IDO1-silenced DC-based therapy against cancer. For this purpose, we prepared an IDO1-targeting siRNA (siIDO1) that was loaded in the YSK12-MEND (YSK12-MEND (siIDO1)) and gene silencing efficiency was evaluated in mouse bone-marrow derived DCs (BMDCs). The silencing efficiency was 70% at a siRNA dose of 20 nM. The efficiency was overwhelmingly superior to commercially available transfection reagents. The DCs with IDO1 silenced by the YSK12-MEND showed a significantly enhanced antitumor effect against E.G7-OVA tumors. Moreover, the level of Treg cells in tumors was decreased in mice that had been treated with the IDO1-silenced DC. The findings reported herein demonstrate that the YSK12-MEND represents an effective delivery system for IDO1-silenced DC based cancer immunotherapy.

## Results and Discussion

### Preparation of siIDO1-loaded YSK12-MEND

**Figure 1:**
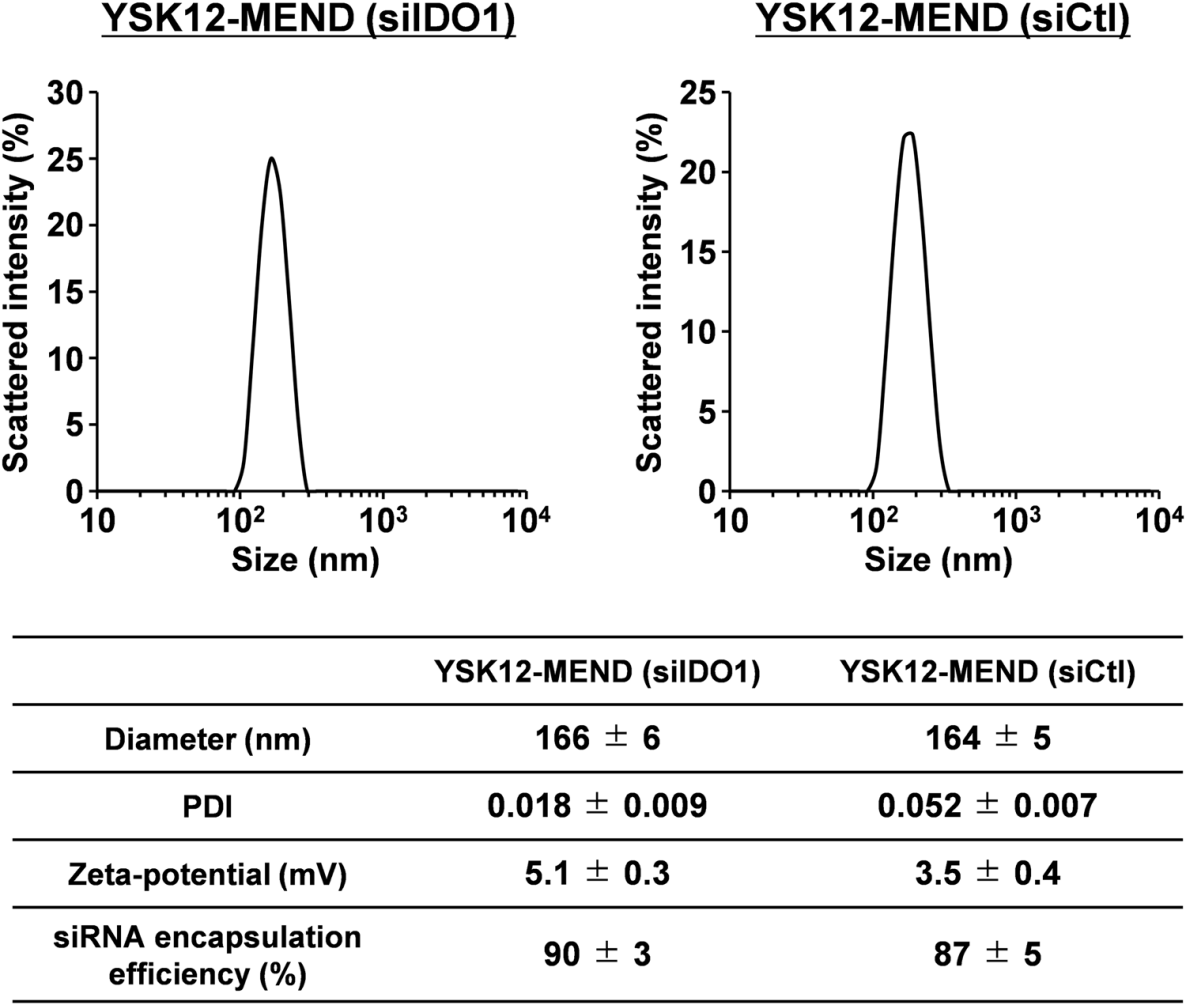
Physical characteristics of the YSK12-MEND (siIDO1 or siCtl). Determination of the particle size distribution, diameter, PDI and zeta-potential of YSK12-MEND (siIDO1 or siCtl) were analyzed with h a ZETASIZER Nano. siRNA encapsulation efficiency was measured by a Ribogreen assay. Data are the mean ± SEM (n = 4–5).

We previously reported on the preparation of siRNA (targeting scavenger receptor class B, member 1 (SR-B1), SOCS1 or GAPDH)-loaded YSK12-MENDs^15,24,25^. We first developed YSK12-MENDs (siIDO1 or siCtl) and assessed their physical properties. The diameter, polydispersity index (PDI), zeta-potential and siRNA encapsulation efficiency between the YSK12-MEND (siIDO1) and the YSK12-MEND (siCtl) were comparable (Fig. 1). The particle size distributions for both YSK12-MENDs were quite sharp and the PDIs were less than 0.1, indicating that they were both homogenous nanoparticles (Fig. 1). Most siRNA molecules are imbedded in the nanoparticle and are not on the surface, because a Ribogreen assay showed that 90% of the siRNA was encapsulated within the nanoparticle. The Ribogreen stain is not able to bind to siRNA molecules inside the nanoparticle. If siRNA molecules were present on the surface of nanoparticle, the fluorescence intensity (FI) would increase substantially, and the siRNA encapsulation efficiency would correspondingly decrease. These results are consistent with previous reports^15,24,25^. The SR-B1 targeted siRNA, SOCS1 targeted siRNA and GAPDH targeted siRNA used in the previous studies were chemically modified siRNA, Stealth siRNA (chemically modified), and Silencer siRNA (chemically unmodified), respectively. The siIDO1 used in the present study was an ON-TARGET plus siRNA (chemically modified). Although the sequence, chemical modification and composition were different, the YSK12-MEND could be similarly used to encapsulate siRNA. This confirms that the YSK12-MEND can be used to encapsulate various types of siRNA.

### Evaluation of IDO1 gene silencing efficiency by the YSK12-MEND in BMDCs

**Figure 2:**
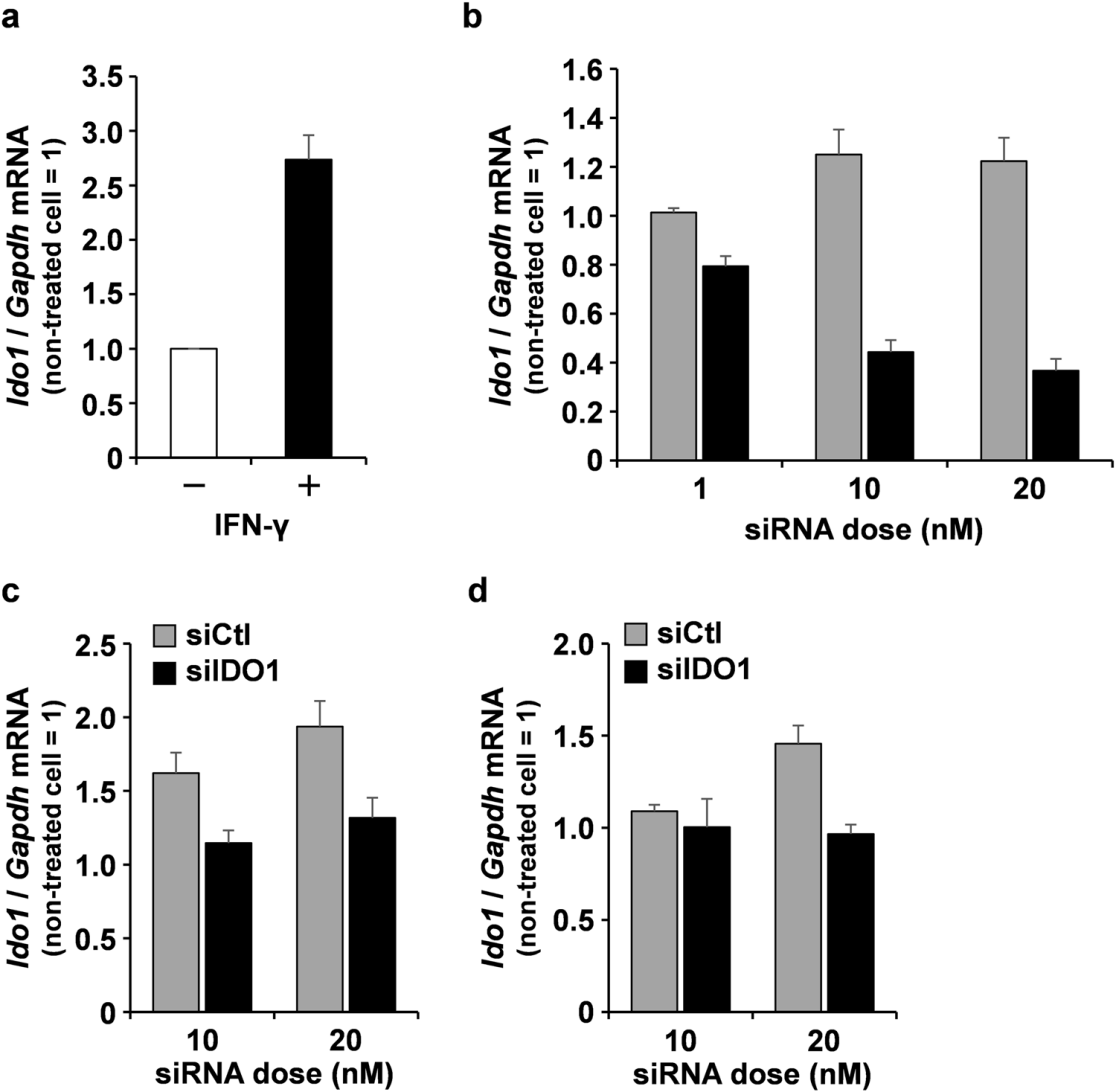
Efficiency of IDO1 gene silencing by the YSK12-MEND in BMDCs. (**a**) The upregulation of IDO1 by an IFN-γ treatment. BMDCs were treated with IFN-γ for 2 h, followed by a 24 h period of culturing. Data are the mean ± SEM (n = 3). (**b**) Gene silencing activity by YSK12-MEND against IDO1 in BMDCs. After the 2 h stimulation with IFN-γ, the BMDCs were transfected with YSK12-MEND (siIDO1 or siCtl) at siRNA doses of 1, 10 and 20 nM. Data are the mean ± SEM (n = 3, **P < 0.01). (**c**) Gene silencing activity by RNAiMAX against IDO1 in BMDCs. After a 2 h stimulation with IFN-γ, the BMDCs were transfected with RNAiMAX (siIDO1 or siCtl) at siRNA doses of 10 and 20 nM. Data are the mean ± SEM (n = 3). (**d**) Gene silencing activity by GeneSilencer against IDO1 in BMDCs. After a 2 h stimulation with IFN-γ, the BMDCs were transfected with GeneSilencer (siIDO1 or siCtl) at siRNA doses of 10 and 20 nM. Data are the mean ± SEM (n = 3). The mRNA levels of IDO1 were measured by quantitative RT-PCR. The value for the non-treated cells was set to 1.

We next examined the extent of IDO1 gene silencing efficiency by the YSK12-MEND (siIDO1) in BMDCs. The expression of IDO1 in BMDCs was very low in the steady state. Thus, the upregulation of the IDO1 gene is needed in order to evaluate the gene silencing effect. An interferon-γ (IFN-γ) treatment upregulated the expression of IDO1 in DCs^14^. BMDCs were treated with IFN-γ for 2 h, followed by a 24 h culture period, and the expression of IDO1 at the mRNA level was measured. The expression of IDO1 in BMDCs treated with IFN-γ was 2.7 times higher than that of control BMDCs (Fig. 2a). After stimulation with IFN-γ, the BMDCs were transfected with the YSK12-MEND (siIDO1 or siCtl) at siRNA doses of 1, 10 and 20 nM. The expression of IDO1 at the mRNA level was measured 24 h after the transfection. The value for the non-treated cells was set to 1. Thus, the values in the y-axis reflect the fold change against the value of non-treated cells. Although IDO1 silencing was not observed in the BMDCs that had been treated with the YSK12-MEND (siCtl), dose-dependent IDO1 silencing was observed in the BMDCs that had been transfected with the YSK12-MEND (siIDO1) (Fig. 2b). The silencing activity was 70% at a siRNA dose of 20 nM. This result indicates that the YSK12-MEND efficiently induces the silencing of the IDO1 gene at a low dose of siRNA in BMDCs. It is anticipated that the expression of IDO1 can be decreased at the protein level, because the result for IDO1 gene silencing at the mRNA level showed similar pattern to that for the protein level (western blotting)^14^. A previous study demonstrated that 2 μg of siRNA was transfected to 10^6^ BMDCs when a commercially available transfection reagent (GeneSilencer) was used^14,26^. In this study, we used 134 ng (20 nM) of siRNA (calculated from the average molecular weight of ON-TARGET plus Mouse Ido1 siRNA-SMART pool) for the transfection of 6 × 10^5^ of BMDCs. We also evaluated the silencing efficiency of commercially available transfection reagents at the same siRNA doses as were used for the YSK12-MEND. As a result, RNAiMAX (Fig. 2c) and GeneSilencer (Fig. 2d) failed to induce IDO1 silencing. This finding suggests that only a small amount of siRNA in the YSK12-MEND was needed to induce sufficient gene silencing compared with commercially available transfection reagents.

**Figure 3:**
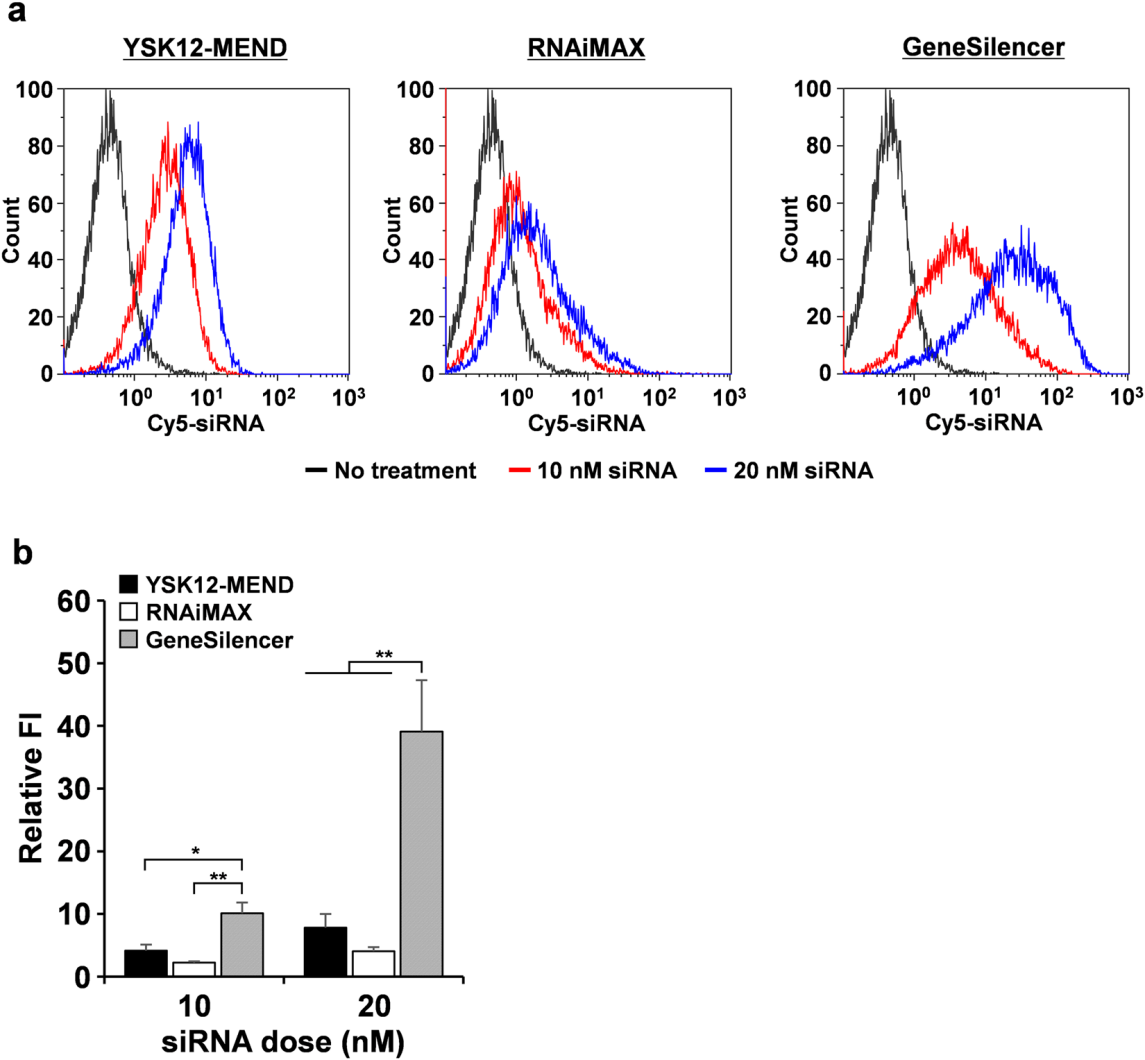
Analysis of cellular uptake by BMDCs. BMDCs were treated with Cy5-siRNA loaded in the YSK12-MEND, RNAiMAX or GeneSilencer at siRNA doses of 10 and 20 nM. After incubation for 2 h (YSK12-MEND and RNAiMAX) or for 4 h (GeneSilencer), the cells were collected and analyzed by flow cytometry. A) Typical histogram. B) Average data of relative FI. Data are the mean ± SEM (n = 3, *P < 0.05, **P < 0.01).

We subsequently compared the efficiency of cellular internalization between the YSK12-MEND and the commercially available transfection reagents. BMDCs were treated with a Cy5-labeled siRNA (Cy5-siRNA) loaded YSK12-MEND, RNAiMAX and GeneSilencer at siRNA doses of 10 and 20 nM, followed by the flow cytometry analysis. In the histograms, a substantial shift was observed in the BMDCs that had been treated with GeneSilencer (Fig. 3a). The relative FI in the BMDCs treated with GeneSilencer was also significantly stronger than those for the YSK12-MEND and RNAiMAX (Fig. 3b). One explanation for this is that the incubation time for the GeneSilencer (4 h: factory-recommended) was longer than those for the YSK12-MEND (2 h) and RNAiMAX (2 h). The uptake activity of the YSK12-MEND was slightly better than the RNAiMAX (Fig. 3b). The difference in cellular uptake between the YSK12-MEND, RNAiMAX and GeneSilencer can also be associated with the particle size. We measured the sizes and PDIs of RNAiMAX and GeneSilencer prepared in the serum-free OPTI-MEM I or PBS (Table S1 in the Supplementary Information). The serum-free OPTI-MEM I was used for the transfection and the YSK12-MEND was prepared in PBS. As a result, the diameter, PDI and zeta-potential of the RNAiMAX were 1268 nm, 0.267, and 26.2 mV in OPTI-MEM I, and 632 nm, 0.193, and 27.5 mV in PBS, respectively. The diameter, PDI and zeta-potential of the GeneSilencer were 1705 nm, 0.297, and 29.8 mV in OPTI-MEM I, and 667 nm, 0.393, and 28.0 mV in PBS, respectively. The diameters, PDIs and zeta-potentials of the RNAiMAX and the GeneSilencer were substantially higher than the corresponding values for the of YSK12-MEND. The size increase of the RNAiMAX reduced cellular uptake efficiency in immune cells^24^. Thus, the low cellular uptake in the case of RNAiMAX can be attributed to its large size. On the other hand, in spite of the large size of the GeneSilencer, it showed a higher cellular uptake than the YSK12-MEND and the RNAiMAX. It is likely that the GeneSilencer had a unique affinity for BMDCs and the high fluorescence intensity that was observed can be attributed to the incorporation of large size particles.

This finding shows that despite the low cellular uptake efficiency, the YSK12-MEND caused an efficient gene silencing. This can be attributed to the efficiency of cytosolic delivery as the result of the strong endosome disruption^15^. Furthermore, it is important top note that the shape of the histogram of the BMDCs incorporating the YSK12-MEND was very similar to the histogram for untreated BMDCs (Fig. 3a). That is, the degree of internalization of the YSK12-MEND was highly homogenous, compared with RNAiMAX and GeneSilencer. The fact that siRNA is uniformly introduced can be a great advantage in terms of the quality of cells used for DC-based therapy.

Consequently, these findings clearly show that the YSK12-MEND is a high potential delivery system for the induction of siRNA into DCs.

### Effect of IDO1 silencing by YSK12-MEND on the phenotypes of BMDCs

**Figure 4:**
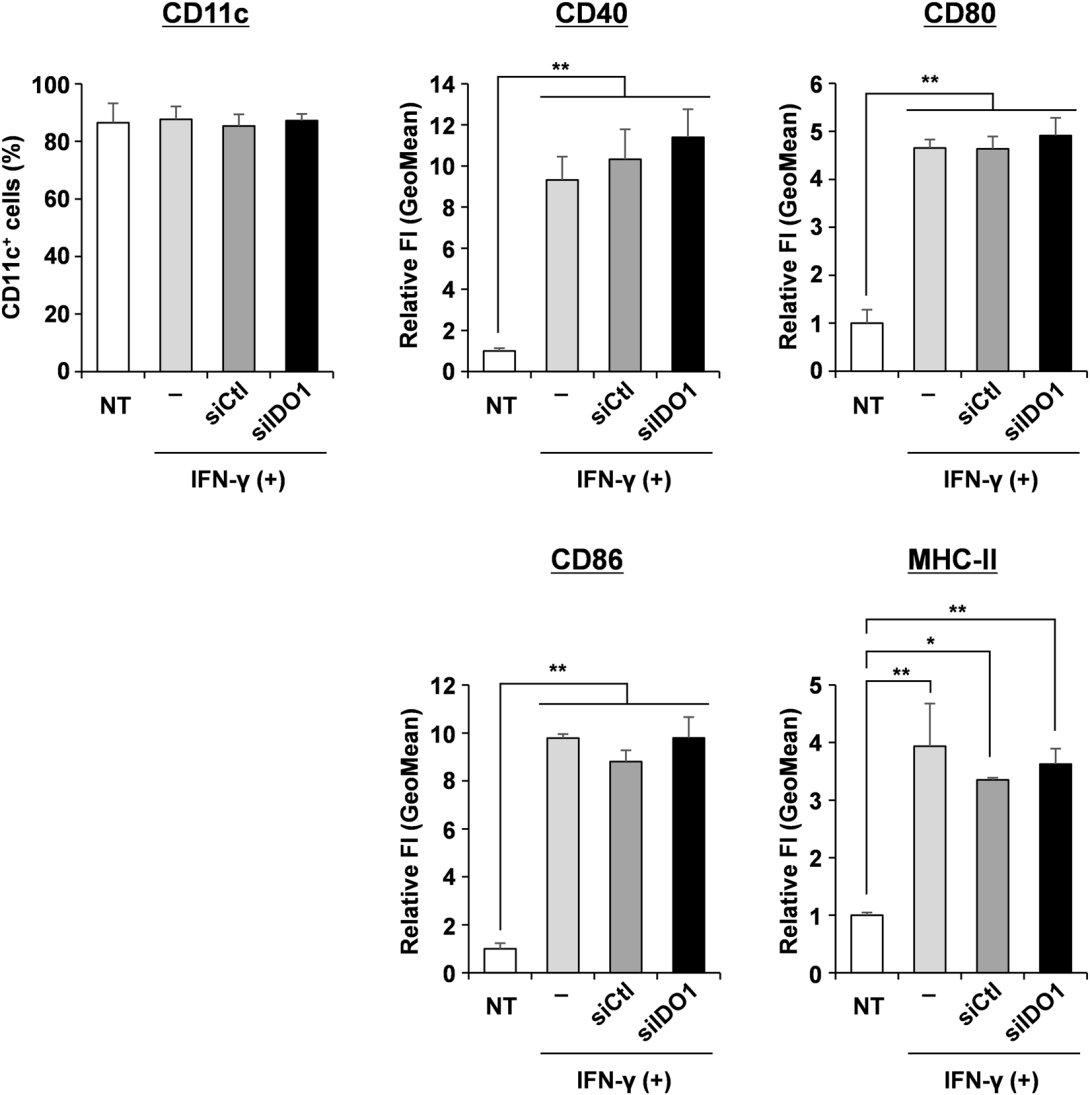
Phenotypes of BMDCs that had been transfected with the YSK12-MEND (siIDO1 or siCtl). After stimulation with IFN-γ for 2 h., the BMDCs were transfected with the YSK12-MEND (siIDO1 or siCtl) at a siRNA doses of 20 nM. The BMDCs were stained with PE anti-mouse CD11c, APC anti-mouse CD40, APC anti-mouse CD80, APC anti-mouse CD86, APC anti-mouse I-A/I-E antibodies or their isotype controls. The FI of the stained BMDCs was measured by flow cytometry. The normalization of GeoMean of FI was performed by dividing by the value of non-treated BMDCs (without IFN-γ and antibodies). Data are the mean ± SEM (n = 3, **P < 0.01, *P < 0.05).

DC maturation induced by activation signals such as adjuvants and cytokines is an important process for the effective priming of T cells. In the case of DC-based therapy, DCs are generally pulsed with antigens and activation signals, leading to DC maturation. Therefore, we analyzed the impact of IDO1 silencing by the YSK12-MEND in the phenotypes of mature BMDCs. After the induction of IDO1 expression by an IFN-γ treatment (stimulation of maturation), the BMDCs were transfected with the YSK12-MEND (siIDO1 or siCtl). After 24 h, the percentage of CD11c^+^ cells and the expressions of CD40, CD80, CD86 and MHC class II (MHC-II) were analyzed by flow cytometry. The CD11c is a major DC marker and the number of CD11c^+^ cells are generally evaluated. The CD40, CD80, CD86 and MHC-II are used as a maturation marker and the change of expression level is generally evaluated. As a control, we used IFN-γ non-treated BMDCs (immature BMDCs). We normalized the obtained FI values (geometric mean: GeoMean) by that for non-treated BMDCs (without IFN-γ and antibodies). As a result, the percentages of CD11c^+^ cells were comparable among the samples, suggesting that the transfection and IDO1 silencing by the YSK12-MEND did not influence the character of BMDCs (Fig. 4). On the other hand, the enhancement of CD40, CD80, CD86 and MHC-II expressions was observed in the IFN-γ treated BMDCs, the IFN-γ + YSK12-MEND (siCtl) treated BMDCs and the IFN-γ + YSK12-MEND (siIDO) treated BMDCs compared with the IFN-γ non-treated BMDCs (Fig. 4). There were no significant differences of the expression of CD40, CD80, CD86 and MHC-II between the IFN-γ treated BMDCs, the IFN-γ + YSK12-MEND (siCtl) treated BMDCs and the IFN-γ + YSK12-MEND (siIDO) treated BMDCs. This suggests that the maturation of BMDCs was due to the IFN-γ treatment, and the transfection and IDO1 silencing by the YSK12-MEND do not appear to affect the phenotypes of mature BMDCs, consistent with a previous report^14^.

### Evaluation of antitumor effect by IDO1-silenced BMDCs by YSK12-MEND

**Figure 5:**
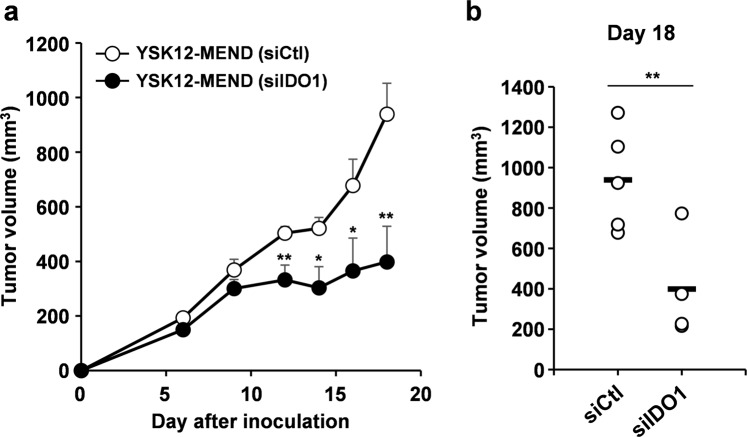
Therapeutic antitumor effect mediated by IDO1-silenced BMDCs by the YSK12-MEND. (**a**) Mice were subcutaneously inoculated with E.G7-OVA cells. On days 4, 8, 11 and 16, the mice were immunized with BMDCs that had been transfected with the YSK12-MEND (siIDO1 or siCtl), and tumor growth was monitored. Data are the mean ± SEM (n = 4–5, **P < 0.01, *P < 0.05). (**b**) Tumor volume on day 18. The circles and bars represent each value and average value, respectively (n = 4–5, **P < 0.01).

To verify the potential of IDO1-silenced DC by the YSK12-MEND for use in cancer immunotherapy, we examined the antitumor effect of the preparation against E.G7-OVA tumors, an immunogenic tumor model. BMDCs were treated with IFN-γ, and subsequently transfected with the YSK12-MEND (siIDO1 or siCtl). The previous result indicates that about 30% of the YSK12-MEND in the transfection medium was taken up by BMDCs^15^. The BMDCs were then pulsed with ovalbumin (OVA) and the SIINFEKL peptide, and were injected into mice bearing E.G7-OVA tumors on day 4, 8, 11 and 16. When gene-silenced DC, sustaining gene silencing is important. In the previous report, the YSK12-MEND maintained a gene silencing effect at protein level for 48 h after the treatment of immune cells^24^. In addition, we used ON-TARGET plus siRNA (Dharmacon) in this study. The siRNAs can have a high capability for sustaining gene silencing, because the siRNAs are chemically modified. The gene silencing of IDO1 was probably considered to last for more than 48 h. On the other hand, the migration of the injected DC to draining lymph nodes was observed at 12 h after the footpads were injected^27^. This fact indicates that gene silencing that can be sustained for at least 12 h or more would be sufficient. In the case of the BMDCs transfected with the YSK12-MEND (siIDO1), the tumor growth was significantly inhibited in comparison with that of the YSK12-MEND (siCtl) at 12 days after the E.G7-OVA inoculation (Fig. 5a). In the group of mice that were treated with BMDCs introduced by the YSK12-MEND (siIDO1), the tumor growth after day 9 was completely suppressed (Fig. 5a), and the difference in tumor volume between the group of mice treated with BMDCs introduced with the YSK12-MEND (siCtl) on day 18 was remarkable (Fig. 5b). The effector cells that killed E.G7-OVA tumor cells can be OVA-specific CTL, because the immunization of OVA induced the OVA-specific CTL and anti-tumor effect against E.G7-OVA tumor^28–33^. The result indicates that BMCDs in which the IDO1 was silenced by the YSK12-MEND enhance the antitumor effect of DC-based therapy against immunogenic tumors.

### Analysis of Treg cells in tumors after IDO1-silenced DC based therapy

**Figure 6:**
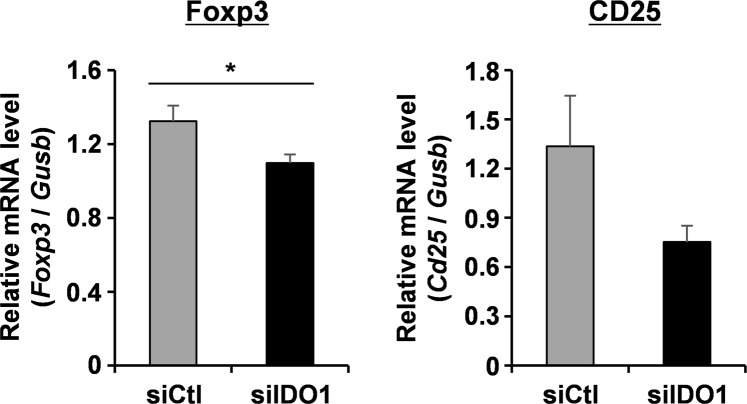
Frequency of Treg cells in E.G7-OVA tumors after IDO1-silenced DC based therapy. Mice were subcutaneously inoculated with E.G7-OVA cells. On days 4, 8, 11 and 16, the mice were immunized with BMDCs that had been transfected with the YSK12-MEND (siIDO1 or siCtl). On day 18, the tumors were collected and the mRNA level of Foxp3 and CD25 were measured by quantitative RT-PCR. The values for PBS treated mice were set to 1. Data are the mean ± SEM (n = 4–5, *P < 0.05).

As pointed out above, one of the negative aspects of IDO1-expressing DCs is the differentiation of naïve T cells to Treg cells^10,19^. In human patients, vaccination with IDO1-positive DCs was reported to result in an enhanced production of Treg cells^34^. To address this issue, we investigated changes in the Treg cell population in E.G7-OVA tumors after treatment with IDO1-silenced BMDCs. Tumor tissues were collected on day 18 of the antitumor experiment (Fig. 5), and the mRNA levels of forkhead box P3 (Foxp3) and CD25 were measured by quantitative RT-PCR. Treg cells are generally identified as Foxp3^+^CD25^+^CD4^+^ cells^35^. The mRNA level of Foxp3 in the E.G7-OVA tumors was significantly decreased by the treatment with BMDC introduced by the YSK12-MEND (siIDO1), compared with that of the YSK12-MEND (siCtl) (Fig. 6). In addition, the mRNA level of CD25 was also reduced on treatment with BMDC introduced by the YSK12-MEND (siIDO1), but the differences were not significant (Fig. 6). These results indicate that IDO1 silencing by the YSK12-MEND (siIDO1) in BMDCs inhibited the increase in the Treg cell population in the tumor microenvironment. It is likely that the reduction of Treg cells enhanced the antitumor effect by IDO1-silenced DC therapy. The expression of IDO1 results in an enhanced level of tryptophan catabolism, leading to tryptophan starvation and an increase in the levels of kynurenines (a product of tryptophan catabolism). Tryptophan starvation upregulates the general control nonderepressible 2 (GCN2) protein kinase, which is responsive to amino acid starvation, resulting in the differentiation of naïve T cells to Treg cells^36^. Kynurenines also bind to the aryl hydrocarbon receptor (AhR) transcription factor that is expressed by naive T cells, leading to differentiation to Treg cells^37^. It is likely that the silencing of IDO1 by the YSK12-MEND reduces the involvement of IDO1-expressing DC treatment in these pathways in the differentiation to Treg cells.

## Conclusion

The findings reported here show that a YSK12-MEND efficiently silenced the IDO1 gene in BMDCs using a low dose of siRNA, and that the IDO1-silenced BMDCs promoted the antitumor effect mediated by DC-based therapy. The YSK12-MEND could be used to homogenously introduce siRNA to BMDCs compared with commercially available transfection reagents, and to induce a sufficient level of gene silencing at siRNA doses which the commercially available transfection reagents were nearly completely ineffective. This procedure is greatly superior to these used in previous studies^14,20^. The enhancement in antitumor effect can be attributed to the reduction in the levels of Treg cells that are produced in the tumor microenvironment. The results indicate that the YSK12-MEND represents a promising system for achieving IDO1-silencing DC-based therapy against cancer. Similar to previous reports, the findings indicate that the use of siRNA for inhibition IDO1 activity in DCs represents an attractive strategy for reducing the levels of Treg cells in the tumor microenvironment. IDO1 is attractive target for inhibiting immune suppression, secondary to immune checkpoint molecules such as programmed death 1 (PD-1). More than 10 clinical trials using IDO1 inhibitors (small-molecule drugs) have been initiated, and a year ago, the future of IDO1 inhibitors looked bright. However, the negative results associated with phase III trials using IDO1 inhibitors (small-molecule drug) that were reported in 2018 have dampened this enthusiasm and several companies have now cancelled, suspended, or downsized phase III trials using small-molecule drugs^38^. The capability of the drug, and not the target (IDO1), might be the problem. The target patients also might not have been suitable. Anyway, it is suggested that new strategy is required instead of small-molecule drugs. IDO1 knockdown by a YSK12-MEND represents an effective strategy for the IDO1 targeted therapy in the future.

## Methods

### Materials

YSK12-C4, (6Z, 9Z, 28Z, 31Z)-19-(4-(dimethylamino)butyl) heptatriaconta-6,9,28,31-tetraen-19-ol, was synthesized as previously described^15^. Cholesterol was obtained from Avanti Polar Lipids Inc. (Alabaster, AL). 1,2-Dimirystoyl-sn-glycerol methoxyethyleneglycol 2000 ether (PEG2000-DMG) was purchased from the NOF Corporation (Tokyo, Japan). RNAiMAX was obtained from Thermo Fisher Scientific (Waltham, MA). GeneSilencer was purchased from Genlantis (San Diego, CA). ON-TARGET plus Mouse Ido1 (15930) siRNA-SMART pool (IDO1-tageted siRNA: siIDO1) and ON-TARGET plus Non-targeting siRNA pool (control siRNA: siCtl) were obtained from Dharmacon (Lafayette, CO). Cy5-siRNA (sense: 5′ -AcAuGAAGcAGcACGACuU(dT*dT)-3′; antisense: 5′-AAGUCGUGCUGCUUC AUGU(dTdT) Cy5-3′, 2′ -OMe are denoted in lower case letters and phoshorothioate linkages are represented by asterisks) was synthesized by BIONEER (Daejeon, Korea). Murine recombinant granulocyte-macrophage colony-stimulating factor (GM-CSF) was purchased from R&D Systems (Basel, Switzerland). Murine recombinant IFN-γ was obtained from PeproTech (Rocky Hill, NJ). Purified anti-mouse CD16/32 (Clone: 93), APC anti-mouse I-A/I-E (clone: M5/114.15.2), APC anti-mouse CD40 (clone: 3/23), APC anti-mouse CD80 (clone: 16-10A1), APC anti-mouse CD86 (clone: GL-1), PE anti-mouse CD11c (clone: N418), their isotype controls, and the 7-AAD Viability Staining Solution were obtained from BioLegend (San Diego, CA). OVA (grade VI) were obtained from SIGMA-Aldrich Co. (St. Louis, MO). Peptide SIINFEKL (MHC class I epitope of OVA) was synthesized by the Toray Research Center (Tokyo, Japan).

### Cells and animal

BMDCs were prepared as reported previously^15,39^. The detail procedure is described in the Supplementary Information. E.G7-OVA cells, a murine lymphoma cell line EL4 expressing OVA, were purchased from the American Type Culture Collection (Manassas, VA) and were cultured in RPMI 1640 medium containing 50 μM 2-mercaptoethanol, 10 mM HEPES, 1 mM sodium pyruvate, 100 U/mL penicillin-streptomycin, 400 μg/mL G418 and 10% fetal bovine serum (FBS).

Female C57BL/6J mice (6–9 weeks old) were purchased from Japan SLC Inc. (Shizuoka, Japan) and maintained under specific pathogen-free conditions. The use of the mice was approved by the Ethics of Pharmaceutical Science Animal Committee of Hokkaido University (approval number: 16-0014). All experiments were performed in accordance with National University Corporation Hokkaido University Regulations on Animal Experimentation.

### Preparation of YSK12-MEND

The YSK12-MEND used in this study was prepared by the tert-butyl alcohol (t-BuOH) dilution procedure^15,24,25^. The detail procedure is described in the Supplementary Information. The lipid composition of the YSK12-MEND was YSK12-C4/cholesterol/ PEG2000-DMG (85/15/1 mol ratio). Although siIDO1 or siCtl were basically used, 10% of the siIDO1 was replaced to Cy5-siRNA when an analysis was done on cellular uptake^24^.

### Measurement of IDO1 gene silencing efficiency

The IDO1 gene silencing activity was evaluated at the mRNA level as reported previously with some modification^15,24,25^. BMDCs (6.0 × 10^5^ cells) were cultured for 2 h in 0.5 mL of serum-free OPTI-MEM I containing 10 ng/mL GM-CSF and 100 U/mL IFN-γ. The medium was then replaced with 0.5 mL of serum-free OPTI-MEM I containing 10 ng/mL GM-CSF and YSK12-MEND (siIDO1 or siCtl) at siRNA doses of 1, 10, and 20 nM, and the resulting BMDCs were incubated for 2 h. RNAiMAX and GeneSilencer reagents were mixed with siRNA solutions following the manufacturer’s instructions. The medium was then replaced with 0.5 mL of serum-free OPTI-MEM I containing 10 ng/mL of GM-CSF and the RNAiMAX or GeneSilencer preparations at siRNA doses of 10 and 20 nM. The BMDCs treated with RNAiMAX and GeneSilencer were incubated for 2 h and 4 h, respectively. After the incubation period, 0.5 mL of culture medium containing GM-CSF was added to the BMDCs, followed by a further incubation for 22 h. After the incubation, the BMDCs were collected and the mRNA expression was evaluated by qRT-PCR. The detail procedure is described in the Supplementary Information. IDO1 levels were calculated by the comparative CT method using GAPDH. The value for the non-treated cells was set to 1.

### Analysis of cellular uptake by BMDCs

The analysis of cellular uptake was performed as reported previously with some modification^24,25^. BMDCs (6.0 × 10^5^ cells) were cultured for 2 h in 0.5 mL of serum-free OPTI-MEM I containing 10 ng/mL of GM-CSF and 100 U/mL of IFN-γ. The medium was then replaced with 0.5 mL of serum-free OPTI-MEM I containing 10 ng/mL of GM-CSF and YSK12-MEND (Cy5-siRNA) at siRNA doses of 10 and 20 nM, and the resulting BMDCs were incubated for 2 h. RNAiMAX and GeneSilencer reagents were mixed with the siRNA solution (Cy5-siRNA) following the manufacturer’s instructions. The medium was then replaced with 0.5 mL of serum-free OPTI-MEM I containing 10 ng/mL of GM-CSF and the RNAiMAX or GeneSilencer preparations at siRNA doses of 10 and 20 nM. The BMDCs that had been treated with RNAiMAX and GeneSilencer were incubated for 2 h and 4 h, respectively. After the incubation period, the cells were collected and washed with 20 U/mL of heparin-PBS. The cells in a PBS containing 0.1% NaN_3_ and 0.5% bovine serum albumin (FACS buffer) were then analyzed by flow cytometry (Gallios, Beckman Coulter, Indianapolis, IN). The Kaluza software program (Beckman Coulter) was used for the data analysis. We normalized the obtained values of FI (GeoMean) by that for non-transfected BMDCs.

### Analysis of surface marker of BMDCs

BMDCs (6.0 × 10^5^ cells) were transfected with the YSK12-MEND (siIDO1 or siCtl) at a siRNA dose of 20 nM as described in the above section. The BMDCs were collected and then dispersed in FACS buffer. The BMDCs were treated with anti-mouse CD16/32 (10 μg/mL) for blocking. After washing, the BMDCs were stained with APC anti-mouse I-A/I-E, APC anti-mouse CD 40, APC anti-mouse CD80, APC anti-mouse CD86, PE anti-mouse CD11c or their isotype controls. The FI of the stained BMDCs was measured by flow cytometry (SH800, Sony, Tokyo, Japan). The data analysis was performed by SH800 software. We normalized the obtained values of FI (GeoMean) by that for non-treated BMDCs (without IFN-γ and antibodies).

### Evaluation of antitumor effect by the IDO1-silenced BMDCs

The evaluation of antitumor was performed as reported previously with some modification^15^. BMDCs (6.0 × 10^5^ cells) were cultured for 2 h in 0.5 mL of serum-free OPTI-MEM I containing 10 ng/mL of GM-CSF and 100 U/mL of IFN-γ. The medium was then replaced with 0.5 mL of serum-free OPTI-MEM I containing a 10 ng/mL solution of GM-CSF and the YSK12-MEND (siIDO1 or siCtl) at an siRNA dose of 20 nM, and the BMDCs were incubated for 2 h. After the 2 h incubation period, 0.5 mL of culture medium containing GM-CSF was trated to the BMDCs. After the 2 h incubation, the BMDCs were washed with culture medium and suspended in fresh culture medium, followed by a further incubation for 2 h. The BMDCs were then pulsed with the SIINFEKL peptide (750 nM) and OVA (50 µg/mL) for 1.5 h. After the incubation, the BMDCs were collected and washed with PBS. The mice were subcutaneously inoculated with 1 × 10^6^ E.G7-OVA cells. On days 4, 8, 11 and 16, the mice were immunized by an injection of 3.0 × 10^5^ mature BMDCs into the hind footpads. Tumor volume (mm^3^) = (major axis × minor axis^2^) × 0.52.

### Analysis of treg cells in E.G7-OVA tumor

In the tumor challenge experiment, the tumors were collected on day 18. Zirconia beads (mixture of 1 mm and 2 mm) and 600 μL of RNAiso Plus (Takara Bio Inc.) were added to the tumors (25 mg). The tumors were homogenized with a Micro Smash homogenizer (MS-100R, TOMY, Tokyo, Japan). After centrifugation, 350 µL of supernatant was collected and mixed with 350 µL of ethanol. The RNA was isolated with a Direct-zol RNA MiniPrep (Zymo Research, Irvine, CA) according the manufacturer’s instructions. The total RNA was then reverse transcribed using a PrimeScript RT reagent Kit with an oligo-dT primer and random 6 mers. Quantitative PCR was carried out on a Light Cycler 480 System in a reaction mixture containing cDNA, with appropriate pairs of primers and the THUNDERBIRD SYBR qPCR Mix. The mRNA levels of Foxp3 and CD25 were calculated by the comparative CT method using β-glucuronidase (Gusb) as an endogenous gene. The values for the PBS treated mice were set to 1. The following primer pairs were used: Foxp3: 5′-TGCAGGGCAGCTAGGTACTTG-3′ (forward); 5′-TCGGAGATCCCCTTTGTCTTATC-3′ (reverse); CD25: 5′-AACCATAGTACCCAGTTGTCGG-3′ (forward); 5′-TCCTAAGCAACGCATATAGACCA-3′ (reverse). Gusb: 5′-GTGGTATGAACGGGAAGCAAT-3′ (forward); 5′-AACTGCATAATAATGGGCACTGT-3′ (reverse).

### Statistical analyses

Comparisons between the two treatments were performed by the unpaired t-test. Statistical analyses of multiple comparisons were performed by one-way ANOVA, followed by the Tukey-Kramer test (Fig. 3b). A P-value of < 0.05 was considered to be significant.

## Supplementary information

Supplementary Information

## Data Availability

The data generated during the current study are available from the corresponding authors on reasonable request.
