# Supplementary material for: The silencing of indoleamine 2,3-dioxygenase 1 (IDO1) in dendritic cells by siRNA-loaded lipid nanoparticles enhances cell-based cancer immunotherapy
Source: Sci Rep. 2019 Aug 5;9:11335. doi: 10.1038/s41598-019-47799-w (PMC6683295; doi:10.1038/s41598-019-47799-w)
Supplement: Supplementary file 1 — Supplementary Information [file 41598_2019_47799_MOESM1_ESM.docx]

**Supplementary Information**

**Title**

The silencing of indoleamine 2,3-dioxygenase 1 (IDO1) in dendritic cells by siRNA-loaded lipid nanoparticles enhances cell-based cancer immunotherapy

**Author**

Rikito Endo^†^, Takashi Nakamura^†*^, Kyoko Kawakami, Yusuke Sato, and Hideyoshi Harashima^*^

Faculty of Pharmaceutical Sciences, Hokkaido University, Kita-12, Nishi-6, Kita-ku, Sapporo 060-0812, Japan

^†^Rikito Endo and Takashi Nakamura contributed equally to this study.

*Correspondence:

Takashi Nakamura

Faculty of Pharmaceutical Sciences, Hokkaido University

Sapporo, Hokkaido 060-0812, Japan

Telephone: +81-11-706-3918, Fax: +81-11-706-3734

E-mail: tnakam@pharm.hokudai.ac.jp

Hideyoshi Harashima

Faculty of Pharmaceutical Sciences, Hokkaido University

Sapporo, Hokkaido 060-0812, Japan

Telephone: +81-11-706-3919, Fax: +81-11-706-3734

E-mail: harasima@pharm.hokudai.ac.jp

**Table S1**

**Diameters and PDIs of the RNAiMAX and the GeneSilencer preparations**

| Name | Preparation buffer | Diameter (nm) | PDI | Zeta-potential (mV) |
| --- | --- | --- | --- | --- |
| RNAiMAX | OPTI-MEM I | 1268±144 | 0.267±0.046 | 26.2±1.3 |
|  | PBS | 632±82 | 0.193±0.012 | 27.5±4.7 |
| GeneSilencer | OPTI-MEM I | 1705±16 | 0.297±0.021 | 29.8±1.1 |
|  | PBS | 667±25 | 0.393±0.034 | 28.0±3.3 |

Data are the mean ± SEM (n = 3).

**Methods**

*Preparation of YSK12-MEND*

The YSK12-MEND used in this study was prepared by the tert-butyl alcohol (t-BuOH) dilution procedure^1-3^. The lipid composition of the YSK12-MEND was YSK12-C4/cholesterol/ PEG2000-DMG (85/15/1 mol ratio). Briefly, a 600 pmol siRNA (siIDO1 or siCtl) solution (200 μL) was slowly added to a 90% (v/v) t-BuOH solution (400 μL) with vortexing. When an analysis was done on cellular uptake, 10% of the siIDO1 was replaced to Cy5-siRNA^1^. The t-BuOH solution contained 425 nmol of YSK12-C4, 75 nmol of cholesterol, and 5 nmol of PEG2000-DMG. The mixed solution was then diluted with 2 mL of citrate buffer (20 mM, pH 6.0) (final concentration of t-BuOH: < 20%). Finally, the residual t-BuOH was replaced with PBS (pH 7.4) by ultrafiltration. The diameters, polydispersity indexes (PDIs) and zeta-potentials of each of the YSK12-MEND preparations were measured in 10 mM HEPES buffer (pH7.4) with a ZETASIZER Nano (ZEN3600, Malvern Instruments Ltd., Malvern, WR, UK). The siRNA encapsulation efficiency and total concentration of siRNA were determined by a Ribogreen assay as described previously^1-3^.

*Preparation of Lipofectamine RNAiMAX (RNAiMAX) and GeneSilencer*

RNAiMAX and GeneSilencer were prepared according the manufacturer’s instructions. The RNAiMAX reagent was diluted in serum-free OPTI-MEM I or PBS. Diluted siRNA in serum-free OPTI-MEM I or PBS was added to the diluted RNAiMAX reagent (1:1 ratio). The mixture was then incubated for 5 min. The GeneSilencer reagent was diluted with serum-free OPTI-MEM I or PBS. A diluted siRNA in serum-free OPTI-MEM I or PBS was added to the diluted GeneSilencer reagent. The mixture was then incubated for 5-30 min. The diameters and PDIs of the RNAiMAX and GeneSilencer preparations were measured with a ZETASIZER Nano instrument. The zeta-potentials were measured in 10 mM HEPES buffer (pH7.4) with a ZETASIZER Nano.

*Culture of mouse BMDCs*

BMDCs were prepared as reported previously^2,4^. Bone marrow cells were collected from mice. The adherent cells were removed and the non-adherent cells were cultured in RPMI1640 medium containing 50 µM 2-mercaptoethanol, 10 mM HEPES, 1 mM sodium pyruvate, 100 U/mL penicillin-streptomycin, 10% FBS and 10 ng/mL GM-CSF (culture medium). Non-adherent cells were removed on days 2 and 4 and adherent cells were cultured in fresh culture medium. On day 6, the non-adherent and loosely adherent cells were used as immature BMDCs. A flow cytometry analysis showed that more than 85% of the BMDCs were CD11c positive.

*Evaluation of IDO silencing activity at the mRNA level*

The evaluation of gene silencing at the mRNA level was performed as reported previously^1-3^. BMDCs (6.0×10^5^ cells) were cultured for 2 h in 0.5 mL of serum-free OPTI-MEM I containing 10 ng/mL GM-CSF and 100 U/mL IFN-γ. The medium was then replaced with 0.5 mL of serum-free OPTI-MEM I containing 10 ng/mL GM-CSF and YSK12-MEND (siIDO1 or siCtl) at siRNA doses of 1, 10, and 20 nM, and the resulting BMDCs were incubated for 2 h. RNAiMAX and GeneSilencer reagents were mixed with siRNA solutions following the manufacturer’s instructions. The medium was then replaced with 0.5 mL of serum-free OPTI-MEM I containing 10 ng/mL of GM-CSF and the RNAiMAX or GeneSilencer preparations at siRNA doses of 10 and 20 nM. The BMDCs treated with RNAiMAX and GeneSilencer were incubated for 2 h and 4 h, respectively. After the incubation period, 0.5 mL of culture medium containing GM-CSF was added to the BMDCs, followed by a further incubation for 22 h. After the incubation, the BMDCs were collected and the RNA isolated with a RNeasy Mini Kit (QIAGEN, Hilden, Germany) according the manufacturer’s instructions. To eliminate DNA contamination, the collected RNA was treated with DNase I (Takara Bio Inc., Shiga, Japan). The RNA was purified by phenol/chloroform extraction and ethanol precipitation. In the case of ethanol precipitation, 1 μL of a glycogen solution (20 mg/mL) was added to the RNA solution. The total RNA was then reverse transcribed using a PrimeScript reverse transcription (RT) reagent Kit (Takara Bio Inc.) with oligo-dT primer and random 6 mers. Quantitative PCR was carried out on a Light Cycler 480 System (Roche Diagnostics, Basel, Switzerland) in a reaction mixture containing cDNA, with appropriate pairs of primers and the THUNDERBIRD SYBR qPCR Mix (TOYOBO Co., Osaka, Japan). IDO1 levels were calculated by the comparative CT method using GAPDH as endogenous housekeeping genes. The value for the non-treated cells was set to 1. The following primer pairs were used: IDO1: 5’-CAAAGCAATCCCCACTGTATCC-3’ (forward); 5’-ACAAAGTCACGCATCCTCTTAAA-3’ (reverse); GAPDH: 5’-AACTTTGGCATTGTGGAAGG-3’ (forward); 5’-GTCTTCTGGGTGGCAGTGAT-3’ (reverse).

**References**

1. Nakamura, T. *et al.* Small-sized, stable lipid nanoparticle for the efficient delivery of siRNA to human immune cell lines. *Sci Rep* **6**, 37849 (2016).

2. Warashina, S. *et al.* A lipid nanoparticle for the efficient delivery of siRNA to dendritic cells. *J Control Release* **225**, 183-191 (2016).

3. Nakamura, T., Yamada, K., Fujiwara, Y., Sato, Y. & Harashima, H. Reducing the Cytotoxicity of Lipid Nanoparticles Associated with a Fusogenic Cationic Lipid in a Natural Killer Cell Line by Introducing a Polycation-Based siRNA Core. *Mol Pharm* **15**, 2142-2150 (2018).

4. Nakamura, T., Moriguchi, R., Kogure, K., Shastri, N. & Harashima, H. Efficient MHC class I presentation by controlled intracellular trafficking of antigens in octaarginine-modified liposomes. *Molecular Therapy* **16**, 1507-1514 (2008).
